# Mottled Duck introductions to South Carolina: The ugly, the bad, and the good?

**DOI:** 10.1002/ece3.8850

**Published:** 2022-04-28

**Authors:** J. Brian Davis

**Affiliations:** ^1^ 5547 Department of Wildlife, Fisheries & Aquaculture Mississippi State University Mississippi State Mississippi USA

**Keywords:** *Anas fulvigula*, conservation introduction, founder populations, Mottled Ducks, South Carolina, translocations

## Abstract

Translocations or other movements of wildlife sometimes accomplish their intended objectives, but unforeseen consequences may arise and disrupt locally adapted ecological communities, restructure or dilute genetic integrity of populations or subspecies of the moved organism, and otherwise negatively influences a species’ long‐term fitness. Two historical populations of Mottled Ducks (*Anas fulvigula*) exist and are endemic to (1) Mexico and the West‐Gulf Coast (*A*. *f*. *maculosa*) regions of the United States and (2) Florida (*A*. *f*. *fulvigula*). From 1975 to 1983, 1285 Mottled Ducks from Florida, Louisiana, and Texas were released to coastal South Carolina, primarily to ultimately establish a legally harvestable population. This movement stirred mixed reactions amid the conservation community. Contemporary information suggests an increasing Mottled Duck population in South Carolina and possibly dispersing into Georgia. Herein, I objectively discuss the potential consequences of this new population per the birds’ evolution, ecology, and management. Ultimately, I suggest that this translocation is a long‐term benefit to the species.

## Graphical abstract

Two historical populations of Mottled Ducks exist and are endemic to (1) Mexico and the West‐Gulf Coastregions of the United States and (2) Florida. Mottled Ducks were moved by conservationists to coastal SouthCarolina in the 1970’s. This third established population has potential evolutionary and ecological outcomes for the species.

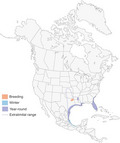

## 1 INTRODUCTION

There is a long history of translocation, a human‐mediated movement and free release of wildlife by humans (Seddon et al., 2012, 2014). Animals may be transported for retention of population viability, assisted colonization, and several other species’ conservation‐related objectives (Evans et al., 2018; Seddon et al., 2014; Tobias et al., 2020). Translocations used as a conservation strategy have greatly increased in recent decades (Bouzat et al., 2009). Approximately 124 species were translocated worldwide from 1900 to 1992 but that increased to 424 species by 2005 (Seddon et al., 2007, 2014). Translocations often strive to reinforce existing populations or reintroduce a species into an area after a local extinction (Seddon et al., 2014; Thévenin et al., 2018). In some cases, a species may be moved beyond its endemic range for other purposes, such as establishing and growing a population of a huntable species, that is, Mottled Ducks (*Anas fulvigula*) in coastal South Carolina.

The intentional movement of organisms, in this case a conservation introduction where founder Mottled Ducks were released into novel environments, is frequently debated in conservation circles, primarily because of uncertainty related to unintended consequences and outcomes, such as potential negative effects on existing local flora and fauna (Fazey & Fischer, 2009; Ricciardi & Simberloff, 2009a, 2009b; Sax et al., 2009; Schlaepfer et al., 2009). Introduced Mallards, for example, have hybridized with indigenous ducks in Hawaii, Australia, New Zealand, and elsewhere (Rhymer & Simberloff, 1996; Wells et al., 2019). Thomas (2011) alternatively challenged some of the potential negative outcomes given the rapid global changes in habitat losses and modifications. Blois et al. (2013) and Thomas (2011) contend that maintaining current ecological communities or restoring them to some pristine condition seems unrealistic given modern‐day environmental and biological changes, part of which are climate induced.

**FIGURE 1:**
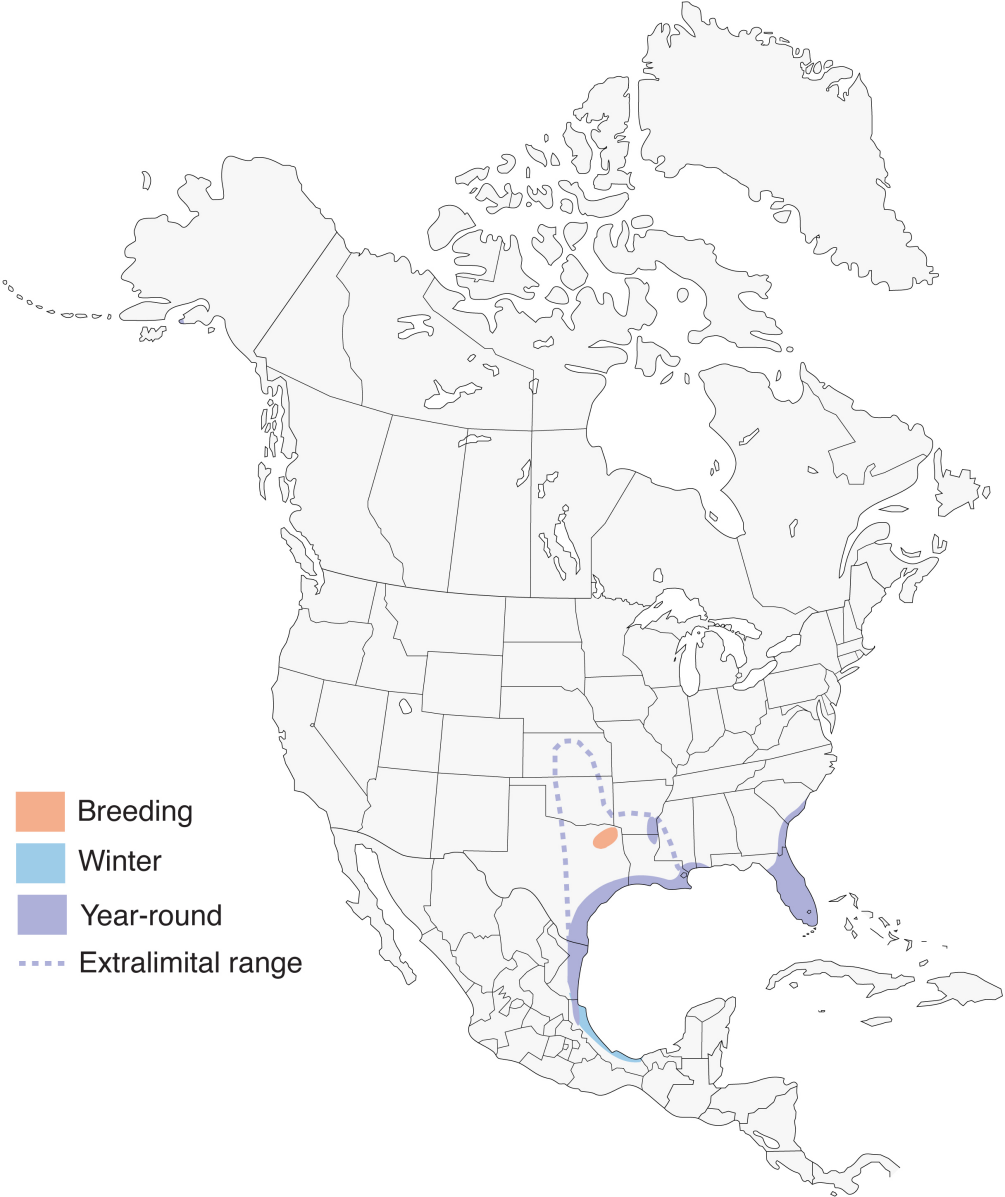
Annual range of the Mottled Duck (*Anas fulvigula*) in North America. Map provided courtesy of All About Birds, The Cornell Lab, March 2022 (https://www.allaboutbirds.org/guide/Mottled_Duck/maps‐range)

The Mottled Duck is an iconic waterfowl species in North America, important to conservationists that include hunters, birders, and general laity (Bielefeld et al., 2020; Lavretsky et al., 2021), and thus is emblematic of such debate. The Mottled Duck is part of an *Anatid* complex which also includes the American Black Duck (*Anas rubripes*), Mallard (*A*. *platyrhynchos*) and the Mexican Duck (*A*. *diazi*) in North America (Bellrose, 1980; Lavretsky et al., 2014; McCracken et al., 2001). The Mottled Ducks’ historic range includes peninsular Florida and coastal areas southwestward from Mobile, Alabama to Veracruz, Mexico (Bellrose, 1980; Bielefeld et al., 2010; Stutzenbaker, 1988). These geographical demarcations separate Mottled Ducks into contemporarily recognized West‐Gulf Coast (*A*. *f*. *maculosa*) and Florida (*A*. *f*. *fulvigula*) populations (Bielefeld et al., 2010; Lavretsky et al., 2021; McCracken et al., 2001) (Figure 1). Recent genetic analyses verify separation of the West‐Gulf Coast and Florida populations (Lavretsky et al., 2014; Weng, 2006). The Mottled Duck is considered one of two non‐migratory dabbling ducks in North America (McCracken et al., 2001), and the bird generally makes daily or seasonal moves of various extent as opposed to being truly migratory (Bellrose, 1980; Dingle, 1996).

## 2 BACKGROUND OF THE RELEASE

In the early 1970s, well‐intentioned waterfowl conservationists in coastal South Carolina wanted Mottled Ducks in their state, primarily for hunting purposes, where no previous populations of the bird existed. From 1975 to 1983, private citizens and the South Carolina Department of Natural Resources (SCDNR) released approximately 1285‐banded birds in the coastal marshes of the Santee River Delta and the Ashepoo, Combahee, Edisto Rivers (ACE) Basin (Kneece, 2016). Of the Mottled Ducks released to South Carolina, 26 originated from the Florida population and 1259 were from Louisiana and Texas (Kneece, 2016). Approximately 107 (8%) banded birds were later identified either through direct or indirect band recoveries from 1975 to 1986 (Kneece, 2016). Only 7 (6%) of these ducks were recovered outside of South Carolina. Hence, Mottled Ducks originally released in South Carolina have demonstrated strong fidelity there. Mottled Ducks from the original South Carolina release now occupy Georgia, although wetland area may be limited and survival rates there are less (i.e., ca. 0.35, males and females) than for Mottled Ducks in Texas‐Louisiana and Florida (Balkcom & Mixon, 2015; Lavretsky et al., 2021; Pollander et al., 2019). Prior to this introduction, there were apparently no records of Mottled Ducks breeding in South Carolina. Presently, Mottled Ducks inhabit at least South Carolina and Georgia, important regions to waterbirds of the South Atlantic Coastal Zone (SACZ; Gordon et al., 1998; Watson & Malloy, 2008). Given the apparent expanded range into Georgia, hereafter I will refer to this new population as SACZ Mottled Ducks (Seyoum et al., 2012) (Figure 1).

At the time of initial release in the 1970s, there was no genetic information available that differentiated West‐Gulf Coast from Florida Mottled Ducks like that available today (Lavretsky et al., 2021; McCracken et al., 2001). Conservationists in the 1970s reconciled that the species would prosper in coastal South Carolina given some similarities in habitats used by Mottled Ducks in coastal Louisiana and Florida (Singleton, 1953; Smith, 1961). The release of Mottled Ducks into South Carolina, however, has created growing awareness in the waterfowl conservation community over potential genetic ramifications (Baldassarre, 2014; Seyoum et al., 2012). There is already concern over declining breeding populations of Mottled Ducks in some portions of its range (e.g., coastal Texas), mostly due to habitat contraction and loss (Wilson, 2007). As such, this release of birds into a novel environment arguably has diverse short‐ and long‐term consequences and outcomes. Three specific negative concerns or hypotheses have been considered, including that currently established SACZ Mottled Ducks will: (1) Hybridize with feral Mallards; in North Carolina, for example, American Black Ducks (*Anas rubripes*) × Mallard introgression has resulted from gene flow through male feral Mallards (Lavretsky et al., 2019; Lawson et al., 2021); (2) hybridize with wild Mallards (Lavretsky et al., 2019), or (3) directly interbreed with the Florida Mottled Duck population, thereby disrupting this latter gene pool that has been generationally distinct (Bielefeld et al., 2010; Lavretsky et al., 2021; Peters et al., 2016). All three of these possibilities could occur and potentially deteriorate the integrity of the Florida Mottled Duck gene pool if subsequent hybrids or pure SACZ Mottled Ducks freely moved into Florida. A fourth and rather unknown result of this release rests with the Mottled Duck's ecological role and interactions with other species in their newly established wetland community (Table 1).

**TABLE 1:** A hypothetical model of potential consequences associated with introducing Mottled Ducks (*n* = 1285) from Florida and the West‐Gulf Coast to coastal South Carolina, 1975–1983

**Sociological‐human dimensions**			
**Positive**	**Unknown**		**Negative**
Waterfowl hunters Bird watchers	N/A		N/A
**Ecological**			
**Positive**	**Unknown**		**Negative**
Perceived "available" waterfowl niche space	Direct competition (food, nests) Interference competition Increased predator awareness (via Mottled Duck presence) to the marsh bird community	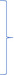	**?**
**Evolutionary**			
**Positive**	**Unknown**		**Negative**
New genetic pool for the species	Degree of hybridization between SACZ Mottled Ducks and feral Mallards Degree of hybridization between SACZ Mottled Ducks and wild Mallards Frequency of pipelining SACZ Mottled Duck genes into Florida Mottled Ducks	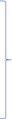	**?**

Note

I refer to this new population as the South Atlantic Coastal Zone (SACZ). Contemporary genetic structure of Mottled Ducks is well understood (e.g., Lavretsky et al., 2021), but potential ecological and future evolutionary consequences remain uncertain, as reflected here.

The current genetic structure of Mottled Ducks and other Mallard‐like species is well documented (Lavretsky et al., 2021; Peters et al., 2016; Weng, 2006). Although it is necessary to briefly overview those dynamics herein, my objective in this paper was to expand the view of the potential outcomes associated with this release (i.e., Table 1). In fact, I offer in the end that this new population is largely a long‐term benefit for the species.

### 2.1 Terminology used herein

For clarity and consistency, I follow the International Union for Conservation of Nature (IUCN) Guidelines for Reintroductions and Other Conservation Translocations (IUCN, 2013). Specifically, I dub this release of Mottled Ducks to coastal South Carolina as a *conservation introduction*, or the intentional movement and release of an organism outside its indigenous range (Hällfors et al., 2014; IUCN, 2013; Seddon, 2010). There are two components of conservation introductions, (a) Assisted colonization, or the intentional movement and release of an organism outside its indigenous range to avoid extinction of populations of the focal species, and (b) ecological replacement, or the intentional movement and release of an organism outside its indigenous range to perform a specific ecological function (IUCN, 2013). Despite these precise definitions, I submit that neither term applies here because genetics of Mottled Ducks, and the species’ ecology for that matter, were not well established at the time of the release. Notwithstanding these definitions, Mottled Ducks were intentionally moved and released to establish a novel population, the potential outcomes of it being the subject of this paper.

## 3 CONTEMPORARY STATUS OF SACZ MOTTLED DUCKS

The population status of SACZ Mottled Ducks is unknown, but available information suggests it has increased since initial release (Kneece et al., 2020). First, based on available hunter harvest data, apparently in only 1 year from 1961 to 1978 were Mottled Ducks harvested in South Carolina (http://flyways.us/regulations‐and‐harvest/harvest‐trends). Since 1979, harvest of Mottled Ducks has been quite variable across years, but 2,750 birds were legally harvested there in 2011 (http://flyways.us/regulations‐and‐harvest/harvest‐trends). Second, Kneece (2016) estimated that 23,000 Mottled Ducks existed in South Carolina by 2008. Collectively, these data suggest an expanded regional population and achieving the original purpose, to grow the new population into a sustainable harvestable one. Population growth of these birds, however, has fueled concern over genetic integrity of the species, particularly that for Florida Mottled Ducks.

### 3.1 Mottled Duck genetics

The West‐Gulf Coast and Florida Mottled Duck populations are as nearly divergent from each other as the Florida Mottled Duck is from the Mallard (Lavretsky et al., 2014; Peters et al., 2016). In fact, Bielefeld et al. (2010) and Callahan (2005) suggested that these Mottled Duck cohorts be designated as separate subspecies. Banding and band recovery data also support the allopatric nature of these populations. For instance, for the thousands of Mottled Ducks banded and recovered, no Mottled Ducks banded in Florida have been recovered in Texas or Louisiana (*n* = 2075 recoveries), and Mottled Ducks banded in Texas and Louisiana have never been recovered in Florida (*n* = 8111 recoveries; Baldassarre, 2014, Peters et al., 2016). More recently, Peters et al. (2016) concluded that the demographic history of the West‐Gulf Coast and Florida Mottled Ducks have been diverging with low levels of gene flow, perhaps in the range of 1–3 migrants per generation, for tens‐of‐thousands of generations. Hence, waterfowl conservationists, particularly in Florida, fear introgressive contamination of Florida Mottled Ducks from the SACZ population.

### 3.2 Aspects of the introduction—The perceived “ugly”

#### 3.2.1 Pollution of Mottled Duck genes from Mallards

Release of captive‐reared Mallards by sportsmen for hunting purposes has occurred for decades in some regions of North America (USFWS, 2013). At least 270,000 captive‐reared Mallards were released annually (as of 2008) on shooting preserves in the United States, and these actions occur today in several states (USFWS, 2013). As North American duck populations declined and hunting opportunities became more restrictive in the 1980s, there was heightened interest in harvesting captive‐reared Mallards on shooting preserves (USFWS, 2013). As much as 50% of captive‐reared Mallards released on Maryland's Eastern Shore, for example, survived a hunting season in the state, free to move among the regulated shooting areas (RSA) and other habitats occupied by wild waterfowl. Some of these feral individuals along the Eastern Shore paired with wild Mallards and Black Ducks (USFWS, 2013). Feral Mallards can survive outside of shooting preserves, and Lavretsky et al. (2019), Lavretsky et al. (2021) recently determined that these birds are impacting genetics of North America's wild Mallard population, with one potential outcome being modified bill morphology as is evidenced in Europe (Söderquist et al., 2014).

A lurking question is whether increased geographic proximity of SACZ Mottled Ducks to feral Mallards exacerbates mating opportunities and subsequent introgressed genes, particularly if they are pipelined into Florida? Mallards have long posed a conservation paradox for other Mallard‐like ducks in North America, especially the American Black Duck because of hybridization or introgression of Mallard alleles into the species (Ankney et al., 1986; Bielefeld et al., 2010; Mank et al., 2004; Peters et al., 2014; Stutzenbaker, 1988; USFWS, 2013). In support of limited hybridization or gene flow, Peters et al. (2014) explained that as much as four times more genetic diversity has likely resulted through gene flow between Mallards and Mottled Ducks, compared to the potential diversity achieved had they been completely isolated though time. An important alternative to this explanation, however, is that introgression of non‐native genes could cause extinction of native genotypes that confer local adaptations with introduction of maladaptive alleles (Quilodrán et al., 2018; Todesco et al., 2016), especially if Mottled Ducks hybridize with feral Mallards. Regarding hybridization between American Black Ducks and Mallards, Lavretsky et al. (2019) regarded such interactions as wasted reproductive effort (Quilodrán et al., 2018) and maladaptive compared to their parentals.

Despite the potential negative implications of maladaptive alleles, Ford et al. (2017) recently estimated low levels (~5%–8%) of hybridization between Mallards and Mottled Ducks in the West‐Gulf Coast, and this value was approximately 9% for Florida birds (Ford et al., 2017; Williams, Brust, et al., 2005; Williams, Fedynich, et al., 2005). Other studies demonstrated little contemporary gene flow among these Mallard‐like species and posited that genetic extinction is unlikely for Mottled Ducks (Lavretsky et al., 2014, 2021; Weng, 2006). Lavretsky et al. (2021), in fact, did not detect any Mottled Duck/feral Mallard hybrids or backcrosses and declared these interactions as currently limited, perhaps because of habitat segregation between the species.

Lastly and concomitant with the genetic evidence, SACZ Mottled Ducks and Mallards do not appear to be pairing and mating. As part of larger studies, systematic surveys of indicated breeding pairs of Mottled Ducks were conducted from March to June, 2011 to 2014 (Kneece, 2016; Shipes, 2014). A total of 5714 Mottled Ducks were either observed as singles, pairs, or as small groups during 524 survey periods on 10 different wetlands. Not once were Mallards observed in these wetlands, nor were any Mottled Ducks observed accompanying Mallards during the surveys.

### 3.3 Aspects of the introduction—The perceived “bad”

#### 3.3.1 Pipelining SACZ Mottled Duck genes into Florida

Another concern among some waterfowl conservationists is related to gene flow and that SACZ Mottled Ducks will introgress with Florida populations, ultimately eroding genetics of the latter (Baldassarre, 2014). Lavretsky et al. (2021) determined that ~98% of genetic signatures of Mottled Ducks in South Carolina were from the West‐Gulf Coast region, and birds initially moved there from Florida have been swamped by the West‐Gulf Coast birds (Lavretsky et al., 2021). Seyoum et al. (2012) reasoned that even if most SACZ Mottled Ducks do not carry Mallard genes, feral or otherwise, the genetic integrity of Florida Mottled Ducks could be compromised via SACZ birds. However, if results of Pollander et al. (2019) are indicative of movements of SACZ Mottled Ducks into Florida, at least presently, these fears may be quelled; for 47 Mottled Ducks (17 males, 30 females) marked with global positioning system (GPS) transmitters at Rhett's Island Georgia, seven of the marked birds moved northward into South Carolina, and only one hatch year male and an after‐hatch‐year female moved to Florida (Pollander et al., 2019).

#### 3.3.2 Vicariance and thinking like an island?

Mottled Ducks obviously occupy habitats on mainland North America. At some point in history, Mottled Ducks in West‐Gulf Coast and Florida became vicariant through a geographic barrier, or by dispersal (peripatry) to establish respective populations (Lavretsky et al., 2014). Florida Mottled Ducks have the lowest levels of recent co‐ancestry with other taxa which indicates long isolation, lower rates of ongoing gene flow, and/or smaller population sizes resulting in greater genetic drift compared to other Mallard‐like ducks (Peters et al., 2016). Unlike the scenario between Mallards and American Black Ducks, where deforestation in northeastern United States has decreased the historical natural barrier between the species, the barrier separating West‐Gulf Coast and Florida Mottled Ducks is arguably much softer, or non‐existent. Yet, mixing of these two populations has been rather absent or very low historically (Lavretsky et al., 2021). Perhaps the distance between suitable habitats for these non‐migratory birds is the simplest explanation. Dispersal and flying abilities of birds are important influences of movements between and among islands and these patterns vary considerably among species (Ando, 2019). The different eco‐regions inhabited by Mottled Ducks are of course mainland, but the nearly non‐existent gene flow between these populations through the millennia harken aspects of island biogeography (Costanzi & Steifetten, 2019; Losos et al., 2009; MacArthur & Wilson, 1967). In other words, it seems these populations settled in their respective geographies, moving around within them, but little between them.

Lack of inter‐regional movements by Mottled Ducks is intriguing, particularly with respect to significant storm events. Approximately 12,000 tropical cyclones occurred globally from 1842–2013 (NOAA, 2013), with a significant number of those reaching the southeastern United States. About 36% of all recorded hurricanes that struck the United States since 1851 made landfall in Florida (http://www.sun‐sentinel.com/news/weather/hurricane/sfl‐hc‐canehistory1,0,3352010.special). Surprisingly, these significant storms apparently did not prompt Florida Mottled Ducks to settle (i.e., founder individuals) coastal South Carolina, a region boasting favorable habitats for the species for the past 400 years, specifically historic rice fields that eventually were converted to managed coastal wetlands (Edgar, 1998; Gordon et al., 1998; Zwank et al., 1989). Perhaps some individuals did move there but were too few to establish populations. Curiosity over the impacts of tropical cyclones on Mottled Ducks has long existed, as molting birds have been killed in storms (Stutzenbaker, 1988). Contemporary information suggests that mortality of Mottled Ducks may be significant in some intensive tropical storms. Ringelman et al. (2021) determined that Hurricane Laura, a Category 4 storm, killed 40% of their radiomarked sample of Mottled Ducks in August 2020. Although Mottled Ducks are considered well‐adapted to tropical storms (Stutzenbaker, 1988), potential negative impacts of storms on population demography (Ringelman et al., 2021) leaves one wondering why individuals over the past four centuries did not disperse to the SACZ, retreating into sometimes less impacted habitats, and ultimately settling as founders. Perhaps the simplest explanation is that 400 years, evolutionarily speaking, is a brief interval. Pollander et al. (2019) found their GPS‐marked Mottled Ducks from Georgia to disperse 52.6–245.8 km. Compare these distances to those between southwest Louisiana (e.g., Grand Chenier) and the central west coast of Florida (e.g., Spring Hill), examples of areas inhabited by the species, being ca.1000 km apart. This evidence suggests that SACZ bird movements into Florida with its potential for introgression will be infrequent for the foreseeable time. Future movements by individuals of this species may hinge on birds’ population sizes, breeding opportunities, and habitat abundance and quality, particularly with respect to climate and other habitat disruptions. But as Lavretsky et al. (2021) concluded, the contemporary integrity of Florida Mottled Ducks is not compromised.

### 3.4 Aspects of the introduction – The “unknown”

Perhaps a more challenging aspect of the introduction to be resolved is the ecological role of SACZ Mottled Ducks in their new wetland community. Given the addition of a large dabbling duck species, how might this influence local niche construction? The Mottled Duck is the only ground‐nesting duck species breeding in coastal South Carolina, but it joins an already rich community of wetland birds (Cely et al., 1993). Shorebirds including Black‐necked Stilt (*Himantopus mexicanus*) and Killdeer (*Charadrius vociferous*) occupy the same wetlands as nesting Mottled Ducks (SCDNR, 2015, Davis *personal observation*). Other marsh dwellers include King (*Rallus elegans*), Clapper (*Rallus crepitans*), and Black Rails (*Laterallus jamaicensis* ssp.), Least Bittern (*Ixobrychus exilis*), Pied‐billed Grebe (*Podilymbus podiceps*), and Purple Gallinule (*Porphyrio martinicus*). These birds to some degree nest in or amid Spartina (*Spartina* spp.) or other vegetation, forming platforms or cups made from grass, sedges, or other marsh plants at varying heights above water (Cely et al., 1993; Kaufman, 1996). Mottled Ducks also nest in dense spartina and other vegetation amid seasonal wetlands (Kneece, 2016; Shipes, 2014). Undoubtedly, some fourth order (Johnson, 1980) resource needs of nesting Mottled Ducks will depart from several co‐existing species. However, how these species partition foraging, and the potential influence of Mottled Ducks on nest clustering and density dependent nest survival (Ringelman et al., 2014) in this avian community are currently unknown but worthy of understanding.

### 3.5 Aspects of the introduction‐‐ The “good”

#### 3.5.1 Novel environments and niche compatibility

Paradoxically, some degree of hybridization between species is good as genetic diversity may introduce variation, novel alleles, and mutations (Alleaume‐Benharira et al., 2006; Frankham, 2005; Garant et al., 2007; Lande & Shannon, 1996). Low rates of gene flow (<2%) for years between two species of Darwin's Finches (*Geospiza fortis* and *G*. *scandens*) apparently enhanced beak morphology and overall fitness of the individuals (Grant & Grant, 2010; Hedrick, 2013; Lamichhaney et al., 2020). In effect, some levels of hybridization can assist adaptations to potentially new niches, and species can expand their climatic ranges resulting from introgression with other species (Krehenwinkel & Tautz, 2013; Stelkens et al., 2014). Peters et al. (2014) posited that introgression of Mallard alleles has helped maintain high genetic diversity in Mottled Ducks, which could benefit the adaptability and survival of the latter. Perhaps an unsettled question is how much gene flow between these populations is acceptable to conservationists? Attempts to safeguard the integrity of Florida Mottled Ducks seems a defensible conservation priority, but also a challenge, relative to evolutionary and ecological processes. Having jurisdiction (i.e., Florida Fish and Wildlife Conservation Commission) over a species with such genetic uniqueness arguably lends itself to the preservation of that gene pool, but at what economic and logistical costs?

Despite the genetic differentiability of Florida and West‐Gulf Coast Mottled Ducks, these birds can only fulfill niche space in current occupied regions so long as there are suitable habitats. Even if the SACZ Mottled Duck genes increased in Florida, the genetic pedigree may change to some extent, but the species should still largely maintain their ecological templet given suitable habitat (Southwood, 1977). Examples in other wetland birds support this notion, such as King Rails (*Rallus elegans*) and Clapper Rails (*R*. *crepitans*) in Atlantic coastal wetlands. Coster et al. (2018) suggested that a King Rail ancestral lineage populated North America and was adapted to freshwater marshes. King Rail have an extensive geographic range whereas Clapper Rails are more specialized and likely predominated salt marshes (Coster et al., 2018). Interestingly, hybridization between species likely occurs in marshes of intermediate salinity at some locations where range overlap occurs (Coster et al., 2018; Eddleman & Conway, 1994; Meanley, 1969; Olson, 1997). Despite the threat of hybridization creating outbreeding depression, reduced fitness, or other consequences (Edmands, 2007; Rhymer & Simberloff, 1996), introgression likely introduced novel genotypes that increase fitness and potentially local adaptations (Coster et al., 2018; Rhymer & Simberloff, 1996). King and Clapper Rails co‐exist in a region of Virginia and introgression is not viewed as deleterious, as Clapper Rails typically do not invade freshwater marshes, thus leaving this habitat type for King Rails (Coster et al., 2018).

Relative to Mottled Ducks, habitats used by Florida birds diverge somewhat from habitats in the West‐Gulf Coast. Florida Mottled Ducks historically have exploited thousands of ponds and irrigation reservoirs associated with ranching, farming, and citrus production inland and other suburban and urban areas (Bielefeld & Cox, 2006). Further south near Lake Okeechobee, Mottled Ducks use storm‐water treatment areas and permanent marshes of the Everglades (Bielefeld, 2008, 2011). The West‐Gulf Coast Mottled Ducks also use freshwater wetlands, ditches, canals, and ricefields, but some birds in the West‐Gulf Coast and South Carolina seek intermediate and brackish wetlands (Baldassarre, 2014; Grand, 1988; Shipes et al., 2015; Zwank et al., 1989). If SACZ Mottled Ducks (i.e., of predominate West‐Gulf Coast origin) move seasonally or otherwise to Florida, it might be that these third‐ and fourth‐ order habitat affinities (Johnson, 1980) create natural niche partitioning among the cohorts of birds, similar to King and Clapper Rails in Virginia.

The evolutionary ecology of animal personalities (Dall et al., 2012; Miranda et al., 2013) may offer some insight into potential interactions between SACZ and Florida Mottled Ducks during their habitat use in Florida. Genetic components of animal personalities can influence resource use of individuals (Miranda et al., 2013; Schielzeth et al., 2011; Van Oers et al., 2004). Cities are evolutionarily novel environments with unfamiliar challenges for wildlife, and urban landscapes are thus ideal systems for understanding how plasticity might promote or hinder adaptation to new environments (Bressler et al., 2020; Shanahan et al., 2013; Sol et al., 2013). In dark‐eyed juncos (*Junco hyemalis*) and Eurasian magpies (*Pica pica*), both demonstrated a protracted breeding season in urban areas, possibly resulting from milder climates or greater food abundances compared with their conspecifics in natural habitats (Bressler et al., 2020; Jerzak, 2001). What remains equivocal for birds generally is whether plasticity is adaptive for urban populations relative to fitness outcomes, and whether urban systems might selectively filter out individuals or species that either do not exhibit behavioral plasticity or show maladaptive plasticity in their new environment (Aronson et al., 2016; Bressler et al., 2020). Human urbanization in parts of Florida is already population dense and increasing, and SACZ Mottled Ducks would seemingly encounter novel environments in suburban and urban areas already occupied by Florida Mottled Ducks (Bielefeld & Cox, 2006). Understanding how the two populations would partition habitats, select mates (Fox, Donelson, et al., 2019), and generally how sexual selection and its relationship with plasticity and adaptations to novel environments (Fox, Donelson, et al., 2019; Fox, Fromhage, et al., 2019) would affect SACZ Mottled Ducks and subsequent progeny would be interesting research ventures.

#### 3.5.2 Habitat composition and future implications of the species

Habitats important to Mottled Ducks are changing throughout their range. Rice fields comprise some of the important habitats used by Mottled Ducks in the West‐Gulf Coast (Grand, 1988; Zwank et al., 1989). Of the six states that produce rice commercially in the United States, area planted has declined fastest in Texas, with an annual average drop of 3.2%, and Louisiana was third (1.7%), from 1995 to 2017 (McBride et al., 2018). Moreover, sea level rise is occurring faster in the Gulf coast of Texas and Louisiana than anywhere else in the United States, where it is rising 7.75 mm/year at Grand Isle, Louisiana and 6.19 mm/year in Galveston, Texas (J. Boon, Virginia Institute of Marine Sciences, unpublished data; https://www.vims.edu/research/products/slrc/index.php). In Florida, the human population is expected to double, to 36 million residents by 2060 (Florida Fish and Wildlife Conservation Commission, unpublished data; https://myfwc.com/media/5478/fwc2060.pdf). Urban sprawl will continue to threaten habitats and further modify native wetland plant communities in Florida (Watson & Malloy, 2008). South Carolina has lost approximately 29% of its wetlands since 1780 (Yarrow, 2009), and wetland losses are especially problematic along coastal South Carolina (Strauss et al., 2014). Moreover, states receiving the greatest levels of human migration from 1995 to 2000 included Georgia, Florida, and South Carolina, which increased the population density of coastal communities by 70% in those states between 1980 and 2003 (Franklin, 2003). Currently, nearly 25% of South Carolina's human population lives along the coastline, with a projected increase to 33% by 2035 (South Carolina Revenues and Fiscal Affairs Office, 2019). These changes to the collective ecoregion of Mottled Ducks, coupled with climate change, will only heighten ecological pressures on the species (Strauss et al., 2014). Thus, I submit that (1) we as conservationists, should strive long‐term to safeguard Mottled Ducks and their habitats wherever suitable resources and opportunities persevere, regardless of genetic differentiation, and (2) this new SACZ population is beneficial in that it provides a third geographically distinct population (albeit West‐Gulf Coast genetics; Lavretsky et al., 2021) of Mottled Ducks in North America.

#### 3.5.3 Are we stalling inbreeding depression?

West‐Gulf Coast and Florida populations differ phenotypically (e.g., plumage and bill color) and are nearly as divergent from each other as they are from other Mallard‐like duck taxa (Lavretsky et al., 2014). With modification to and foreseeable future loss of suitable habitats along coastal Carolina and in Florida, perhaps the most ecologically lucrative outcome of this translocation is that potential consequences of inbreeding depression have been delayed? Inbreeding depression is the mating of close relatives (Wright, 1922) and an artifact of it is expression of deleterious recessive alleles (Roff, 2002; Szulkin & Sheldon, 2007) and a trend toward genome‐wide homozygosity (Keller & Waller, 2002; Szulkin & Sheldon, 2008). Accumulation of deleterious mutations can subsequently reduce individual fitness (Opatová et al., 2016). Fortunately, there are potentially positive outcomes relative to inbreeding depression via individual dispersal. First, Opatová et al. (2016) studied effects of inbreeding on Zebra Finch sperm characteristics and inbred males had more abnormal spermatozoa and lower sperm velocity than outbred males maintained under the same conditions. Hence, dispersal of individuals from one population into another can increase the heterozygosity of a population and minimize breeding among close relatives (Hamilton & May, 1977; Opatová et al., 2016; Szulkin & Sheldon, 2008). Second, the Greater Prairie Chicken (*Tympanuchus cupido pinnatus*) has declined throughout its range in North America, in part due to issues with inbreeding depression (Bouzat et al., 2009). Through conservation intervention because of declining populations, birds were moved from other populations in Midwestern United States and translocated to southeastern Illinois (Bouzat et al., 2009). Ultimately, the Illinois populations benefitted through enhanced genetic diversity, detectable at both nuclear and mitochondrial DNA levels a decade post‐release (Bouzat et al., 2009). These success stories may translate to future Mottled Duck genetics and population dynamics.

#### 3.5.4 Was future genetic rescue unintentionally established?

Certainly, with no vision or intention of “genetic safeguarding” over 40 years ago, waterfowl conservationists may have unknowingly created a new genetic island of Mottled Ducks in the SACZ with long‐term benefits (Bouzat et al., 2009). There is no contemporary evidence of inbreeding depression in any of the Mottled Ducks populations (Lavretsky et al., 2021), but potential habitat loss and degradation throughout the birds’ range may be buffered by this novel SACZ population reservoir as future securement of the species. Some SACZ individuals will undoubtedly disperse southward into Florida, but there may be spatial habitat limitations if resources decline there. Regardless, I hypothesize that most of the SACZ Mottled Ducks will remain in South Carolina, as this represents the greatest extent of wetland habitat potentially benefitting the species in that part of the eastern seaboard (Gordon et al., 1989, 1998). Our understanding of Mottled Duck resource needs in South Carolina is only beginning (Kneece, 2016; Shipes, 2014; Shipes et al., 2015). However, the bird has an affinity for managed wetlands (Shipes et al., 2015), as do other dabbling ducks in coastal South Carolina (Gordon et al., 1998). Most managed impoundments in this region are located on state or federal lands, or on private lands that have progressively protected the resources with perpetual conservation easements (https://www.ducks.org/press‐room/news‐releases/du‐films‐the‐ace‐basin). Approximately 87,816 ha of private lands in the ACE Basin of South Carolina, for example, were permanently protected as of spring 2019 (Ducks Unlimited, Inc., *unpublished data*). These wetlands are buffered against urban expansion and degradation or outright loss and provide resource havens for Mottled Ducks in this ecoregion.

As counterintuitive as it may seem, this new SACZ population could provide a genetic reservoir for future establishment, or re‐establishment of the species, in habitats deemed suitable in North America. If some Mottled Ducks from the SACZ did move into Florida because of density‐dependent breeding conditions or for other reasons, competition among individuals remaining in the native habitats (i.e., Florida) could be relaxed and absolute fitness of Mottled Ducks from SACZ could be enhanced (Uecker et al., 2014), with little compromise to the Florida population. Generally, this third pool of Mottled Ducks may be a hedge‐bet for future species security.

Biologically significant structuring in some wetland birds can occur over small geographic distances, as is evidenced in the Hawaiian gallinule (*Gallinula galeata sandvicensis*) on O’ahu (van Rees et al., 2018). Habitat connectivity (Taylor et al., 1993) can ameliorate many risk factors and allow physically disjunct populations to persist in a network (Crooks & Sanjayan, 2006; Macdonald & Johnson, 2001), or even as a metapopulation of interconnected habitats (Doleman, 2012; Hanski, 1999; Smith & Green, 2005; van Rees et al., 2018). In this light, SACZ Mottled Ducks are proximal to Florida, but at the same time the species demonstrates reluctance to move great distances, thus I hypothesize that population structuring will likely be maintained, but the limited gene flow that may occur could actually benefit both populations, with little fear of genetic homogenization to the Florida birds.

In closing, a primary concern for species viability is how availability of quality habitats influences population size and integrity. With continued climate change, anthropogenic challenges to habitats in Florida and the West‐Gulf Coast, and sea level rise in all parts of the birds’ range, SACZ Mottled Ducks may one day be fundamentally vital to the conservation of the species. The benefits of genetic monitoring (Lavretsky et al., 2021), and studies on the ecology of the species in the SACZ (Kneece, 2016; Shipes, 2014; Shipes et al., 2015) position the Mottled Duck as a species by which to evaluate future moves or translocations of itself or other Aves, if and when warranted.

## CONFLICT OF INTEREST

I have none to declare.

## AUTHOR CONTRIBUTIONS

**J. Brian Davis:**Conceptualization (lead); Writing – original draft (lead); Writing – review & editing (lead).

## Data Availability

Being a Review article, I did not use original data in the manuscript.
